# Comparison of commercially available media for hepatic differentiation and hepatocyte maintenance

**DOI:** 10.1371/journal.pone.0229654

**Published:** 2020-02-27

**Authors:** Yukiko Toba, Sayaka Deguchi, Natsumi Mimura, Ayaka Sakamoto, Kazuo Harada, Kazumasa Hirata, Kazuo Takayama, Hiroyuki Mizuguchi

**Affiliations:** 1 Laboratory of Biochemistry and Molecular Biology, Graduate School of Pharmaceutical Sciences, Osaka University, Osaka, Japan; 2 Laboratory of Hepatocyte Regulation, National Institutes of Biomedical Innovation, Health and Nutrition, Osaka, Japan; 3 Laboratory of Applied Environmental Biology, Graduate School of Pharmaceutical Sciences, Osaka University, Osaka, Japan; 4 PRESTO, Japan Science and Technology Agency, Saitama, Japan; 5 Global Center for Medical Engineering and Informatics, Osaka University, Osaka, Japan; 6 Integrated Frontier Research for Medical Science Division, Institute for Open and Transdisciplinary Research Initiatives (OTRI), Osaka University, Osaka, Japan; University of Tampere, FINLAND

## Abstract

Human hepatocytes are essential materials in pharmaceutical researches. Not only primary human hepatocytes (PHH) but also human iPS cell-derived hepatocyte-like cells (human iPS-HLCs) are expected to be applied as materials for pharmaceutical researches. To date, several culture media have been developed for culturing human hepatocytes. However, there have been no reports comparing these media to determine which is most suitable for culturing human hepatocytes. In this study, we compared five commercial media (Hepatocyte Culture Medium (HCM), HepatoZYME-SFM, Cellartis Power Primary HEP Medium, DMEM/F12, and William’s E Medium (WEM)) to determine which is most suitable for culturing PHH and human iPS-HLCs. In hepatic differentiation of human iPS cells (day 14–25 of differentiation), albumin (ALB) and urea secretion abilities and CYP2C9, CYP2C19, and CYP3A4 activities were the highest when using HCM or WEM. During maintenance of human iPS-HLCs, ALB and urea producing abilities and CYP2C9, CYP2C19, and CYP3A4 activities were the highest when using HCM. Importantly, we found that human iPS-HLCs cultured in HCM were maintained for 3 weeks or more without impairment of their hepatic functions. These results suggest that it is necessary to select an optimal medium for hepatic differentiation and maintenance of human iPS-HLCs. In the case of PHH culture, there was little difference in hepatic functions among the five media. However, the CYP2C9, CYP2C19, and CYP3A4 activities were the highest when using HCM and WEM. In conclusion, it is important to select the optimal medium for specific application when carrying out pharmaceutical researches using human hepatocytes.

## Introduction

Most of drugs are detoxified in the liver. Therefore, human hepatocytes are essential materials for drug toxicity tests in pharmaceutical researches. Primary human hepatocytes (PHH) are often used as a human liver model for drug discovery. Because PHH retain the activities of drug metabolizing enzymes and transporters, *in vitro* assay using PHH has been performed to predict human pharmacokinetics and hepatotoxicities. However, major drawbacks include the limited supply of PHH and the functional deterioration of PHH due to long-term culture. Human induced pluripotent stem cell-derived hepatocyte-like cells (iPS-HLCs) have liver-specific functions and can be supplied on an unlimited basis. We have improved the hepatic differentiation method by transducing transcription factors, using a three-dimensional (3D) spheroid culture system, and modulating the DNA epigenetic status [1–3]. However, the hepatic functions of human iPS-HLCs are still lower than those of PHH.

Various media suitable for the culture of human hepatocytes have been developed. The Hepatocyte Culture Medium (HCM) Bullet Kit (Lonza) is commercially available as a suitable medium for the maintenance culture of PHH. HepatoZYME-SFM (Thermo Fisher Scientific, HepatoZYME) is a serum-free medium for the long-term maintenance of hepatocyte phenotypic phenotypes including the active and inducible forms of cytochrome P450 (CYP). Cellartis Power Primary HEP Medium (Cellartis, Primary HEP) is optimized for long-term maintenance of PHH. Using Primary HEP, the functional CYP activities in PHH have been sustained for 28 days. Dulbecco’s Modified Eagle Medium / Nutrient Mixture F-12 (Thermo Fisher Scientific, DMEM/F12) is a combined medium consisting of DMEM and Ham’s F-12 medium. DMEM/F12 is widely used as a basal medium for supporting the growth of many mammalian cells and stem cells. Xu et al. showed that DMEM/F12 can be used to maintain human embryonic stem cells in an undifferentiated state [4]. William’s E Medium, no phenol red (Thermo Fisher Scientific, WEM) was developed in 1974 as a reduced serum-supplemented medium for long-term cell culture of adult rat hepatocytes [5]. Using WEM, PHH that were cultured for 14 days showed CYP1A2 and CYP3A4 activities equivalent to those at 24 hr after seeding. Several groups have successfully cultured PHH using these five media [6–9]. In addition, HCM, HepatoZYME, and WEM are widely used for culturing human iPS-HLCs [10–13]. As described above, there are many types of medium for human hepatocytes. However, the most suitable medium for culturing of PHH and human iPS-HLCs has not fully been examined.

In this study, we compared these five media (HCM, HepatoZYME, Primary HEP, DMEM/F12 and WEM) to examine which is most suitable for the culture of human iPS-HLCs and PHH. We measured the ALB secretion levels and CYP activities in human iPS-HLCs and PHH.

## Materials and methods

### Hepatocyte culture media

Human iPS-HLCs and PHH were cultured with following five culture mediums.

Hepatocyte Culture Medium Bullet Kit^™^ (Lonza, HCM);

HepatoZYME-SFM (Thermo Fisher Scientific, HepatoZYME);

Cellartis Power Primary HEP Medium (Cellartis, Primary HEP);

DMEM/F12 (Thermo Fisher Scientific) supplemented with Primary Hepatocyte Maintenance Supplements (Thermo Fisher Scientific, DMEM/F12);

William’s E Medium, no phenol red (Thermo Fisher Scientific) supplemented with Primary Hepatocyte Maintenance Supplements (Thermo Fisher Scientific, WEM).

## Human ips cells

The human iPS cell line, Tic (obtained from the JCRB Cell Bank: JCRB Number JCRB1331), was maintained on a feeder layer of mitomycin C-treated mouse embryonic fibroblasts (MEF, Merck Millipore) with ReproStem medium (ReproCELL) supplemented with 10 ng/ml FGF2 as we previously reported [14].

## Hepatic differentiation

For each human iPS cell line used in hepatic differentiation, all differentiated cells were constantly removed by manual collection with a pipette. Before the initiation of hepatic differentiation, human iPS cells were dissociated into clumps by using Dispase II (Roche) and plated onto BD Matrigel Basement Membrane Matrix Growth Factor Reduced (BD Biosciences). The differentiation protocol for the induction of definitive endoderm cells, hepatoblast-like cells, and HLCs was based on our previous report with some modifications [15]. Briefly, in the definitive endoderm differentiation, human iPS cells were cultured for 4 days in RPMI1640 medium (Sigma-Aldrich), which contained 100 ng/ml Activin A (R&D Systems), 1×GlutaMAX, and 1×B27 Supplement Minus Vitamin A (Thermo Fisher Scientific). For the induction of hepatoblast-like cells, the definitive endoderm cells were cultured for 5 days in RPMI1640 medium (Sigma-Aldrich) which contained 20 ng/ml BMP4 (R&D Systems), 20 ng/ml FGF4 (R&D Systems), 1×GlutaMAX, and 1×B27 Supplement Minus Vitamin A. To perform the hepatic differentiation, the hepatoblast-like cells were cultured for 5 days in RPMI1640 medium (Sigma-Aldrich), which contained 20 ng/ml HGF, 1×GlutaMAX, and 1×B27 Supplement Minus Vitamin A. Finally, the cells were cultured for 11 days in various hepatocyte culture medium with 20 ng/ml oncostatin M (OsM).

## Real-time RT-PCR

Total RNA was isolated from human iPS cells and their derivatives using ISOGENE (NIPPON GENE). cDNA was synthesized using 500 ng of total RNA with a Superscript VILO cDNA synthesis kit (Thermo Fisher Scientific). Real-time RT-PCR was performed with SYBR Green PCR Master Mix (Thermo Fisher Scientific) using a StepOnePlus real-time PCR system (Thermo Fisher Scientific). The relative quantitation of target mRNA levels was performed by using the 2^-ΔΔCT^ method. The values were normalized by those of the housekeeping gene, *glyceraldehyde 3-phosphate dehydrogenase* (*GAPDH*). PCR primer sequences (described in Table A in S1 File) were obtained from PrimerBank (https://pga.mgh.harvard.edu/primerbank/).

## ALB and urea secretion

The culture supernatants, which were incubated for 24 hr after fresh medium was added, were collected and analyzed by Enzyme-Linked Immuno Sorbent Assay (ELISA) to determine their levels of ALB secretion. A Human Albumin ELISA Quantitation Set was purchased from Bethyl Laboratories. ELISA was performed according to the manufacturer’s instructions. The amount of ALB secretion was calculated according to each standard followed by normalization to the protein content per well. Protein content was measured using Pierce BCA Protein Assay Kit according to the manufacturer’s instructions.

The culture supernatants, which were incubated for 24 hr after fresh medium was added, were collected and analyzed for the amount of urea production. Urea measurement kits were purchased from BioAssay Systems. The experiment was performed according to the manufacturer’s instructions. The amount of urea secretion was calculated according to each standard followed by normalization to the protein content per well. Protein content was measured using Pierce BCA Protein Assay Kit according to the manufacturer’s instructions.

## Immunocytochemistry

To perform the immunocytochemistry, the human iPS cell-derived cells were fixed with 4% paraformaldehyde (PFA) in PBS for 10 min. After blocking the cells with PBS containing 10% FBS, 1% bovine serum albumin (BSA), and 1% Triton X-100 for 45 min, the cells were incubated with a primary antibody (described in Table B in S1 File) at 4°C overnight, and then with a secondary antibody (described in Table B in S1 File) at room temperature for 1 hr.

## Fluorescence-activated cell sorting (FACS) analysis

To obtain single-cell suspensions, the human iPS cell-derived cells were dissociated using Dispase II (Sigma-Aldrich) and Collagenase (FUJIFILM Wako) for 45 min. Single-cell suspensions of the human iPS cell-derived cells were fixed with 4% PFA at 4°C for 10 min, and then incubated with the primary antibody (described in Table C in S1 File), followed by the secondary antibody (described in Table C in S1 File). Analysis was performed on an MACSQuant Analyzer (Miltenyi Biotec).

## Primary human hepatocytes (PHH)

Plateable cryopreserved human hepatocytes (lots HC10-10; XenoTech) were used in this study. The vials of hepatocytes were rapidly thawed in a shaking water bath at 37°C; the contents of each vial were emptied into prewarmed OptiTHAW Hepatocyte Isolation Kit (XenoTech) and the suspension was centrifuged at 100 *g* for 5 min at room temperature. The hepatocytes were seeded at 2.5x10^5^ cells/cm^2^ in OptiPLATE Hepatocyte Plating Media (XenoTech) on 24-well Corning^®^ BioCoat^™^ Collagen I-coated Microplates (Corning). The medium was replaced with various culture media 6 hr after seeding. The hepatocytes, which were cultured 90 hr after plating the cells, were used in the experiments.

## LC-MS/MS

Human iPS-HLCs and PHH were cultured with medium containing 10 μM phenacetin (PHE, Cambridge Isotope Laboratories), 5 μM midazolam (MDZ, FUJIFILM Wako), 10 μM dicrofenac (DIC, FUJIFILM WAKO), or 50 μM *S*-mephenytoin (S-MP, Toronto Research Chemicals). The metabolites of each substrate are acetaminophen (APAP), 1’-hydroxymidazolam (OHMDZ), 4'-hydroxydicrofenac (OHDIC), and 4’-hydroxy-S-mephenytoin (OHSMP). After the treatment with substrates, the supernatant was collected at 2 hr, and then immediately mixed with two volumes of acetonitrile (FUJIFILM Wako). Samples were filtrated with AcroPrep Advance 96-Well Filter Plates (Pall Corporation) for 5 min at 1,750 *g*, and then the supernatant was analyzed by UPLC-MS/MS to measure the concentration of metabolites according to each standard curve. UPLC analysis was performed using an Acquity UPLC (Waters) and MS/MS was performed on a Q-Premier XE (Waters). The mass spectrometer was set to the multiple-reaction monitoring (MRM) mode and was operated with the electrospray ionization source in positive ion mode. MRM transitions (*m/z* of precursor ion / *m/z* of product ion) for APAP, OHMDZ, OHDIC, and OHSMP were 152.0/110, 342.2/203.1, 312.2/166.8, and 235.2/149.9, respectively. For each transition, the cone voltage and collision energy were set at 28 V, 28 eV (APAP), 40 V, 26 eV (OHMDZ), 24 V, 60 eV (OHDIC), and 30 V, 18 eV (OHSMP). The dwell time for each MRM transition was set at 100 milliseconds. LC separations were carried out at 40°C with an Acquity UPLC BEH C18 column, 1.7 μm, 2.1 X 50 mm (Waters). The mobile phase was delivered at a flow rate of 0.5 ml/min using a gradient elution profile consisting of solvent A (0.1% formic acid/distilled water) and solvent B (acetonitrile). The initial composition of the binary solvent was 10% B from 0 to 0.5 min. Solvent B was increased from 10% to 100% over 2.0 min. The composition of solvent remained for 1.0 min at 100% B. Ten μl of sample solution was injected into the column. The concentrations of each metabolite were calculated according to each standard followed by normalization to the protein content per well. Protein content was measured using Pierce BCA Protein Assay Kit according to the manufacturer’s instructions.

## Results

### Screening of an optimal culture medium for hepatic differentiation of human ips cells

**Fig 1:**
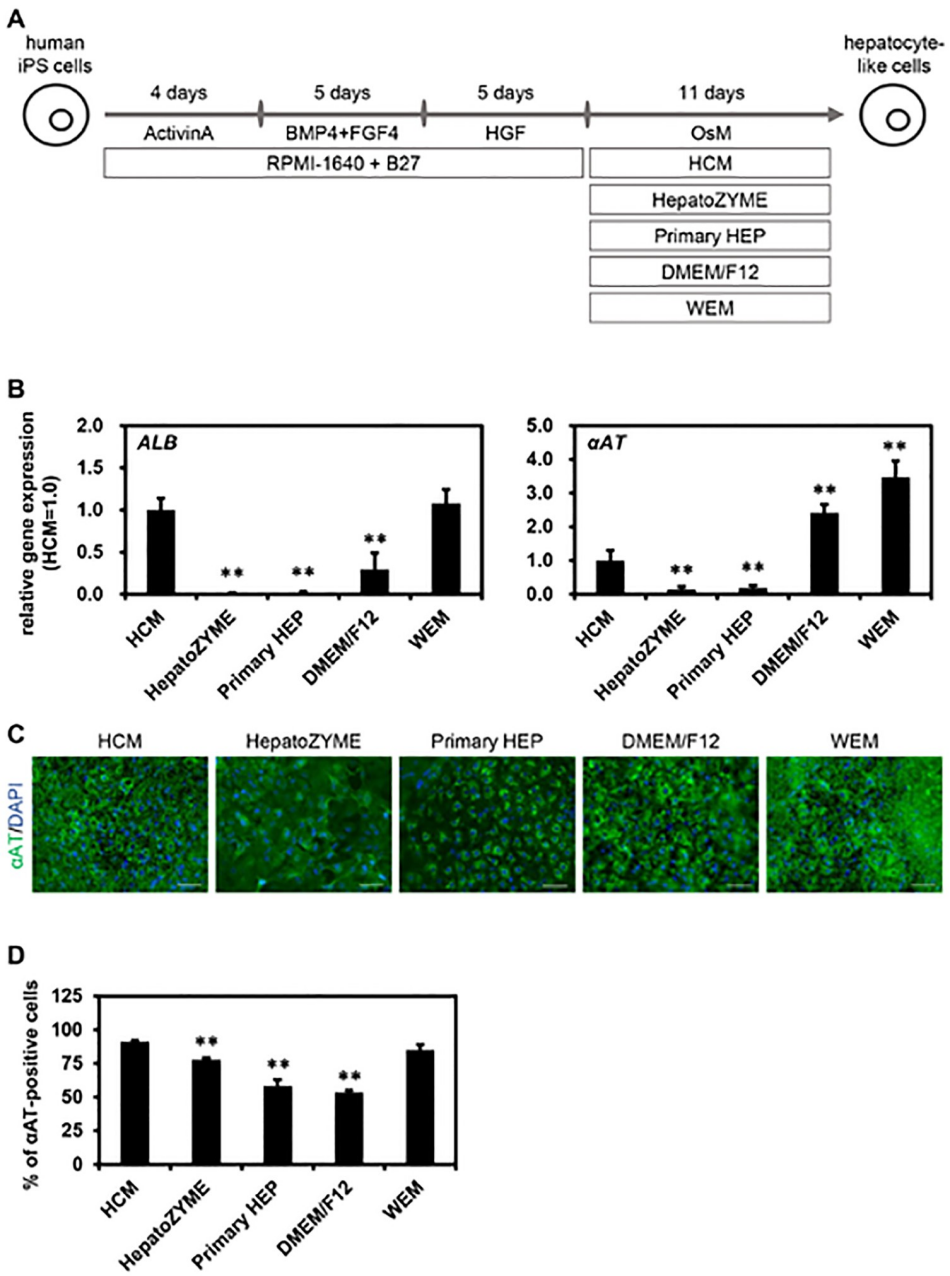
Screening of an optimal culture medium for hepatic differentiation of human iPS cells. (**A**) The screening procedure is presented schematically. Details of the hepatic differentiation procedure are described in the Materials and Methods section. (**B**) The gene expression levels of hepatocyte markers (*ALB* and *αAT*) were examined by real-time RT-PCR. The gene expression levels of human iPS-HLCs cultured with HCM were taken as 1.0. (**C**) The protein expression levels of a hepatoblast marker (αAT (green)) in human iPS-HLCs were examined by immunocytochemical analysis. Nuclei were counterstained with DAPI (blue). The scale bars represent 50 μm. (**D**) The percentages of human iPS-HLCs positive for a hepatocyte marker (αAT) were examined by FACS analysis. Results are shown as the mean ± SD (*n* = 3). Statistical significance was evaluated by one-way ANOVA followed by Dunnet’s test post-hoc tests (compared with “HCM” group). **p < 0.01.

**Fig 2:**
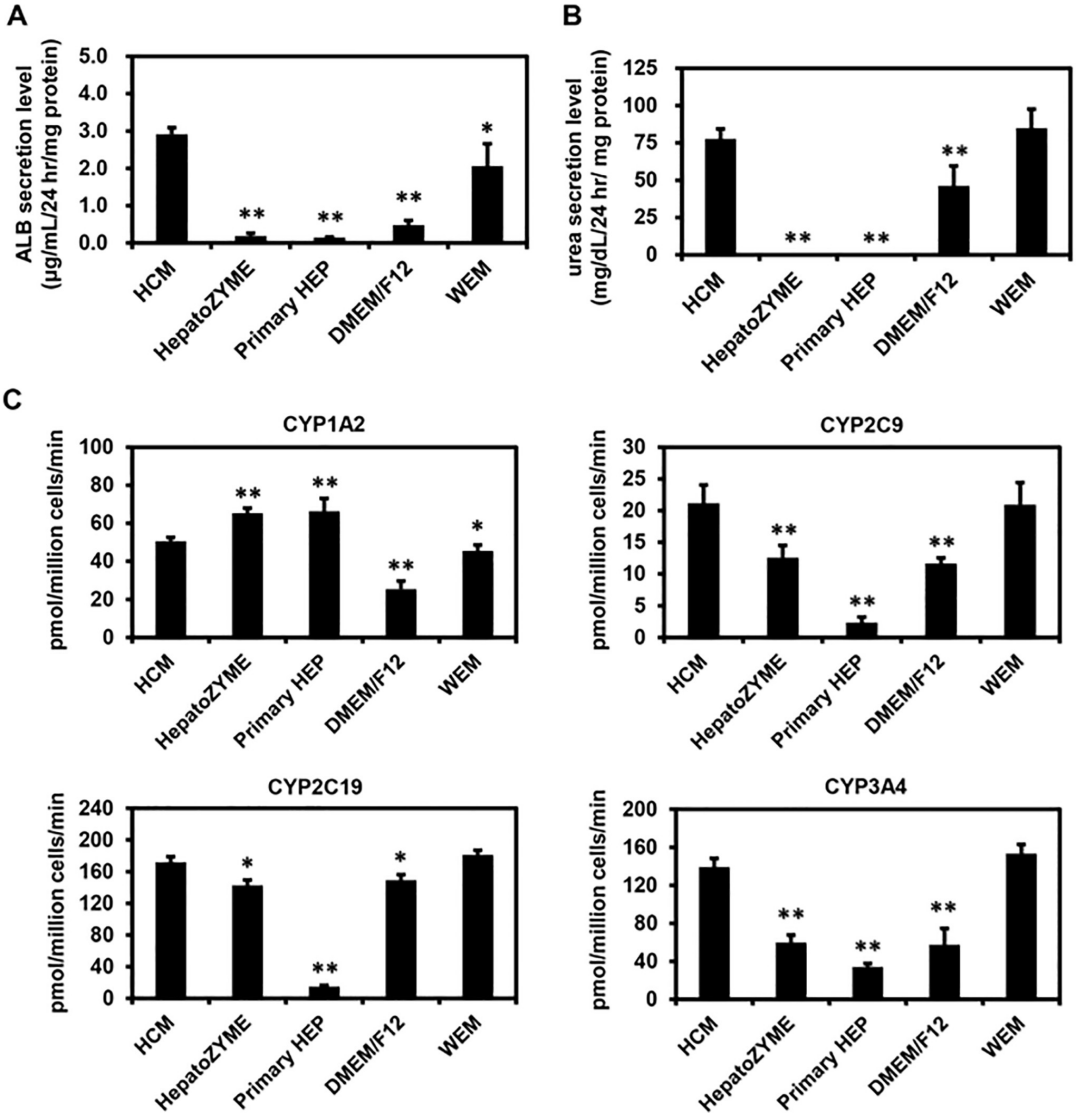
Functional analysis of human iPS-HLCs differentiated with various culture media. Human iPS cells were differentiated into HLCs according to the protocol described in Fig 1A. (A, B) The ALB (**A**) and urea (**B**) secretion capacities in human iPS-HLCs were examined. (**C**) The CYP1A2, CYP2C9, CYP2C19, and CYP3A4 activities in human iPS-HLCs were examined using LC-MS/MS. Results are shown as the mean ± SD (*n* = 3). Statistical significance was evaluated by one-way ANOVA followed by Dunnet’s test post-hoc tests (compared with “HCM” group). *p < 0.05, **p < 0.01.

First, we examined which of the media were suitable for hepatic differentiation of human iPS cells. Human iPS-HLCs were generated according to the protocol shown in Fig 1A. Human iPS cell-derived hepatic progenitor cells (day 14) were cultured with the various media until day 25 of differentiation. There was little difference in the gene expression levels of hepatocyte markers (*ALB* and *αAT*) among human iPS-HLCs cultured with HCM, DMEM/F12, or WEM (Fig 1B). The gene expression levels of hepatocyte markers (*ALB* and *αAT*) in human iPS-HLCs were significantly decreased by using HepatoZYME or Primary HEP (Fig 1B). These results suggest that neither HepatoZYME nor Primary HEP was a suitable medium for the hepatic differentiation of human iPS cells. The human iPS-HLCs cultured with HCM, DMEM/F12, or WEM were positive for αAT and HNF4α (Fig 1C). To examine the hepatic differentiation efficiency, the percentages of hepatocyte marker (αAT) in human iPS-HLCs were examined (Fig 1D). The percentages of αAT-positive cells were the highest when using HCM or WEM. We also performed functional analysis of human iPS-HLCs differentiated with various culture media. The ALB (Fig 2A) and urea (Fig 2B) secretion capacities in human iPS-HLCs were the highest when using HCM or WEM. In addition, CYP2C9, CYP2C19, and CYP3A4 activities in human iPS-HLCs were the highest when using HCM or WEM (Fig 2C). Taken together, these results indicated that HCM and WEM were the most suitable media for hepatic differentiation of human iPS cells.

### Screening of an optimal culture medium for maintenance of human ips-hlcs

**Fig 3:**
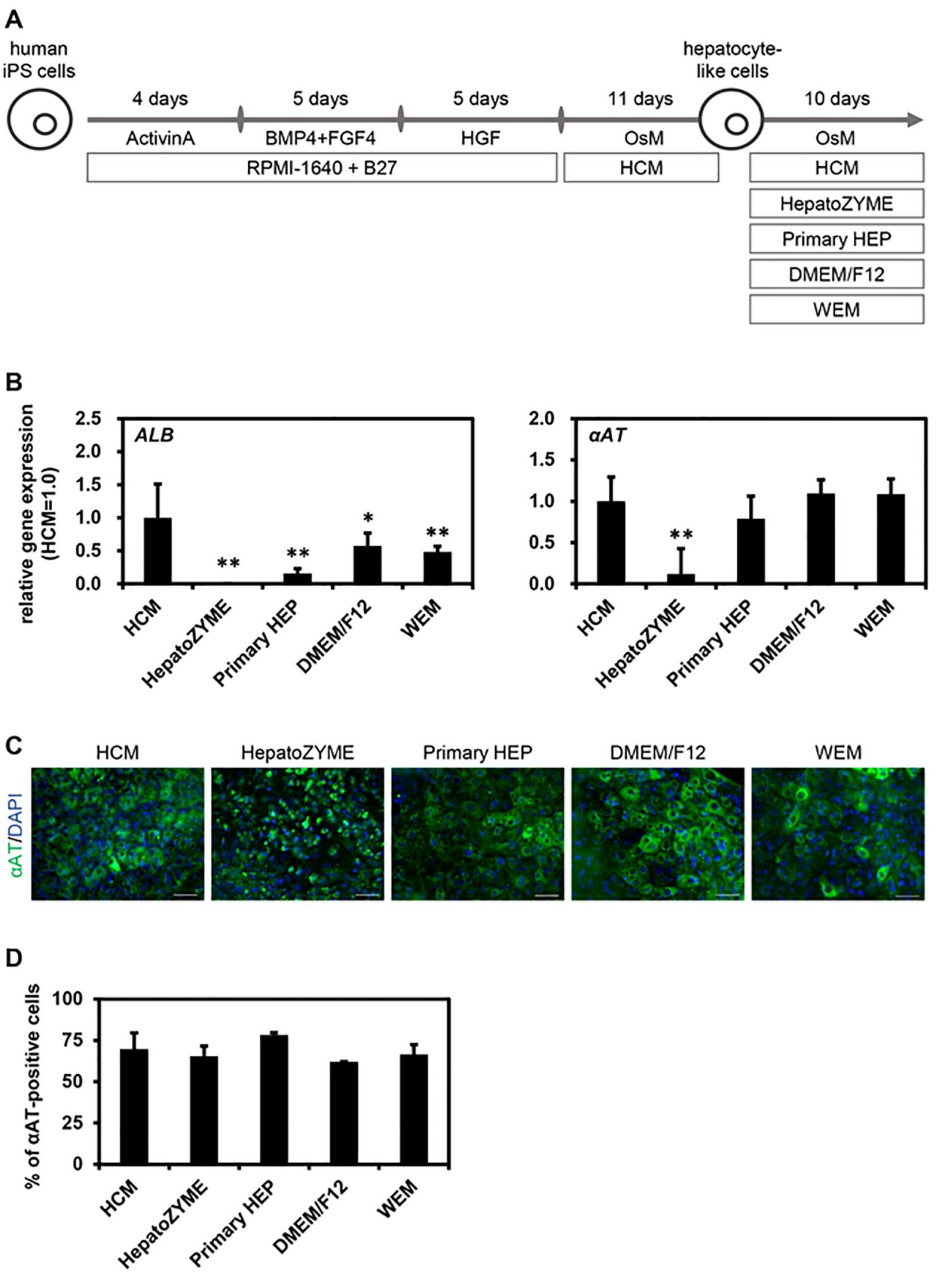
Screening of an optimal culture medium for maintenance of human iPS-HLCs. (**A**) The screening procedure is presented schematically. Details of the hepatic differentiation procedure are described in the Materials and Methods section. Human iPS-HLCs were cultured with various culture media for 10 days. (**B**) The gene expression levels of hepatocyte markers (*ALB* and *αAT*) were examined by real-time RT-PCR. The gene expression levels of human iPS-HLCs cultured with HCM were taken as 1.0. (**C**) The protein expression levels of hepatoblast markers (αAT (green)) in human iPS-HLCs were examined by immunocytochemical analysis. Nuclei were counterstained with DAPI (blue). The scale bars represent 50 μm. (**D**) The percentages of human iPS-HLCs positive for a hepatocyte marker (αAT) were examined by FACS analysis. Results are shown as the mean ± SD (*n* = 3). Statistical significance was evaluated by one-way ANOVA followed by Dunnet’s test post-hoc tests (compared with “HCM” group). *p < 0.05, **p < 0.01.

**Fig 4:**
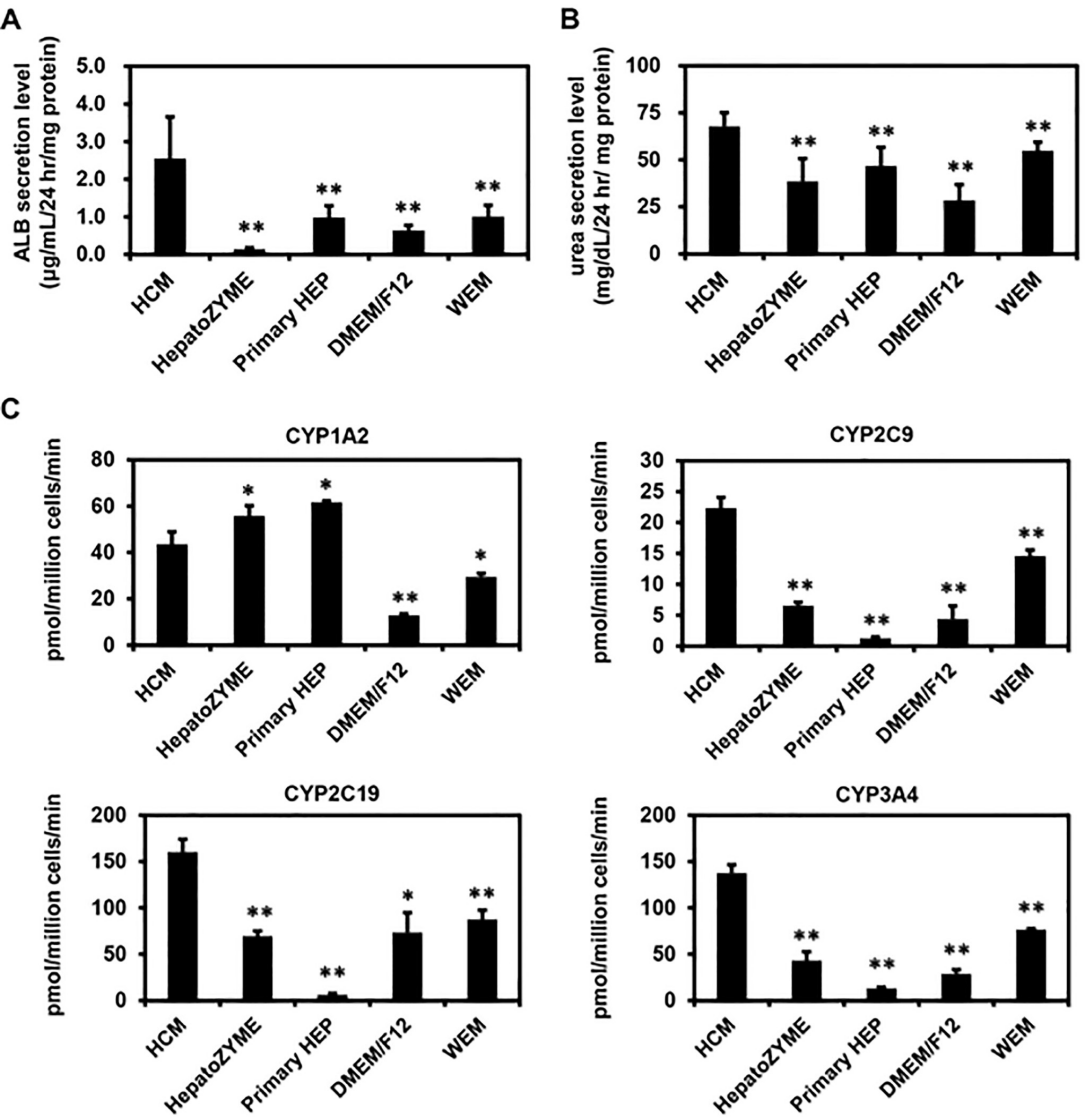
Functional analysis of human iPS-HLCs maintained with various culture media. Human iPS cells were differentiated into HLCs according to the protocol described in Fig 3A. Then, human iPS-HLCs were cultured with various culture media for 10 days. (**A**, **B**) The ALB (**A**) and urea (**B**) secretion capacities in human iPS-HLCs were examined. (**C**) The CYP1A2, CYP2C9, CYP2C19, and CYP3A4 activities in human iPS-HLCs were examined using LC-MS/MS. Results are shown as the mean ± SD (*n* = 3). Statistical significance was evaluated by one-way ANOVA followed by Dunnet’s test post-hoc tests (compared with “HCM” group). *p < 0.05, **p < 0.01.

Next, we examined which of the medium would be the most suitable for the maintenance of human iPS-HLCs. Human iPS-HLCs were generated according to the protocol shown in Fig 3A. Human iPS-HLCs were cultured with various media for 10 days (from day 25 to day 35 of differentiation). There was little difference in the gene expression levels of hepatocyte markers (*ALB* and *αAT*) among human iPS-HLCs cultured with HCM, DMEM/F12, or WEM (Fig 3B). The gene expression levels of *ALB* in human iPS-HLCs were significantly decreased by using HepatoZYME (Fig 3B). These results suggest that HepatoZYME was not a suitable medium for maintenance of human iPS cells. The human iPS-HLCs cultured with HCM, Primary HEP, DMEM/F12, or WEM were positive for αAT and HNF4α (Fig 3C). To examine the hepatic differentiation efficiency, the percentages of hepatocyte marker (αAT) in human iPS-HLCs were examined (Fig 3D). There was little difference in the percentage of αAT-positive cells among the human iPS-HLCs maintained with different media. We also performed a functional analysis of human iPS-HLCs maintained with various culture media. The ALB (Fig 4A) and urea (Fig 4B) secretion capacities in human iPS-HLCs were the highest when using HCM. In addition, CYP2C9, CYP2C19, and CYP3A4 activities in human iPS-HLCs were the highest when HCM was used (Fig 4C). Taken together, our findings showed that HCM was the most suitable medium for maintenance of human iPS-HLCs.

### Long-term culture of human iPS-HLCs

**Fig 5:**
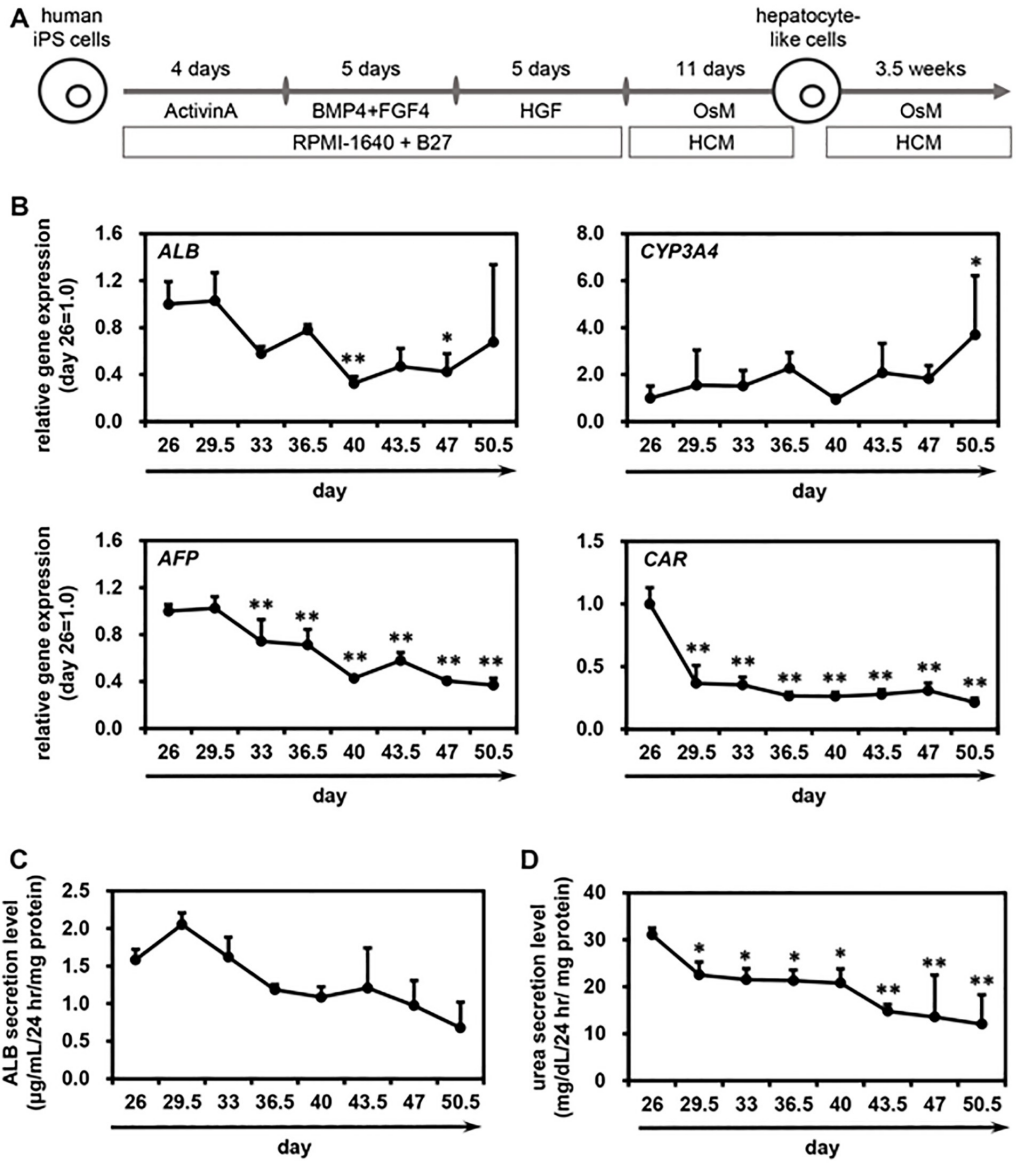
Long-term culture of human iPS-HLCs. (**A**) The procedure for screening is presented schematically. Details of the hepatic differentiation procedure are described in the Materials and Methods section. Human iPS-HLCs were cultured with HCM. (**B**) Temporal gene expression levels of hepatocyte markers (*ALB*, *CYP3A4*, *AFP*, and *CAR*) were examined by real-time RT-PCR. The gene expression levels of human iPS-HLCs (at day 25 of differentiation) cultured with HCM were taken as 1.0. (**C**, **D**) Temporal ALB (**C**) and urea (**D**) secretion capacities in human iPS-HLCs were examined. Mean ± SD (*n* = 3). Statistical significance was evaluated by one-way ANOVA followed by Dunnet’s test post-hoc tests (compared with “day 26” group). *p < 0.05, **p < 0.01.

As described above, HCM was the most suitable medium for maintenance of human iPS-HLCs. Next, therefore, we examined whether the hepatic functions of human iPS-HLCs cultured with HCM could be maintained for a long period. For this purpose, we cultured iPS-HLCs with HCM until day 50.5 (Fig 5A), then examined the temporal gene expression levels of hepatocyte markers (*ALB*, *CYP3A4*, *AFP* and *CAR*) (Fig 5B). The gene expression levels of *ALB* and *CYP3A4* in human iPS-HLCs were not largely changed during the long-term culture. The gene expression levels of *AFP* and *CAR* in human iPS-HLCs were gradually decreased during the long-term culture. We also confirmed that the ALB (Fig 5C) and urea (Fig 5D) secretion abilities of human iPS-HLCs were not largely changed during the long-term culture. These results suggest that human iPS-HLCs can be cultured and maintained for at least additional 25 days by using HCM.

### Screening of an optimal culture medium for primary human hepatocytes

**Fig 6:**
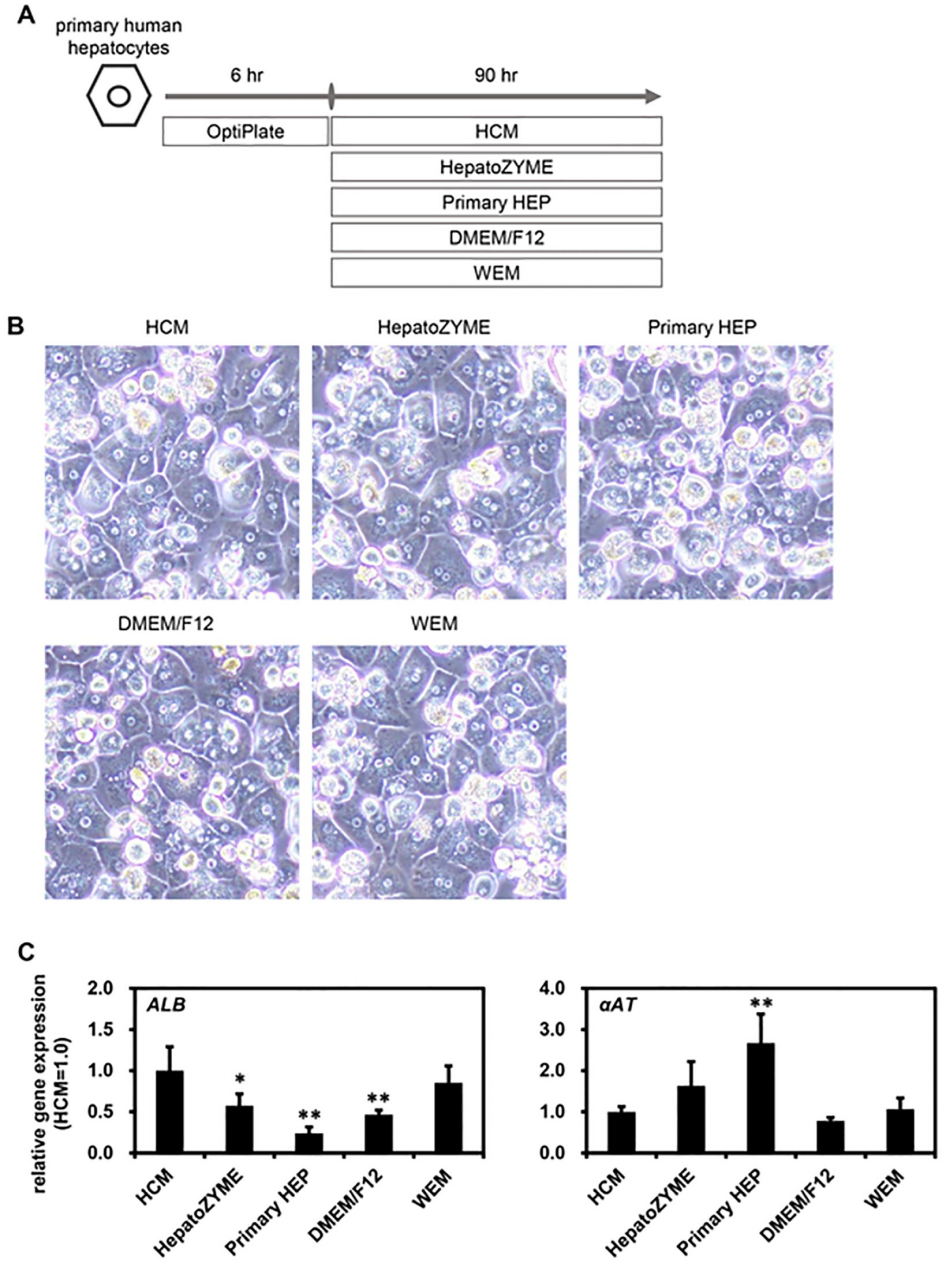
Screening of culture medium for maintenance of primary human hepatocytes. (**A**) The screening procedure is presented schematically. Details of the PHH culture procedure are described in the Materials and Methods section. PHH were cultured with various culture media for 90 hr. (**B**) The phase images of PHH are shown. (**C**) The gene expression levels of hepatocyte markers (*ALB* and *αAT*) were examined by real-time RT-PCR. The gene expression levels of PHH cultured with HCM were taken as 1.0. Results are shown as the mean ± SD (*n* = 3). Statistical significance was evaluated by one-way ANOVA followed by Dunnet’s test post-hoc tests (compared with “HCM” group). *p < 0.05, **p < 0.01.

**Fig 7:**
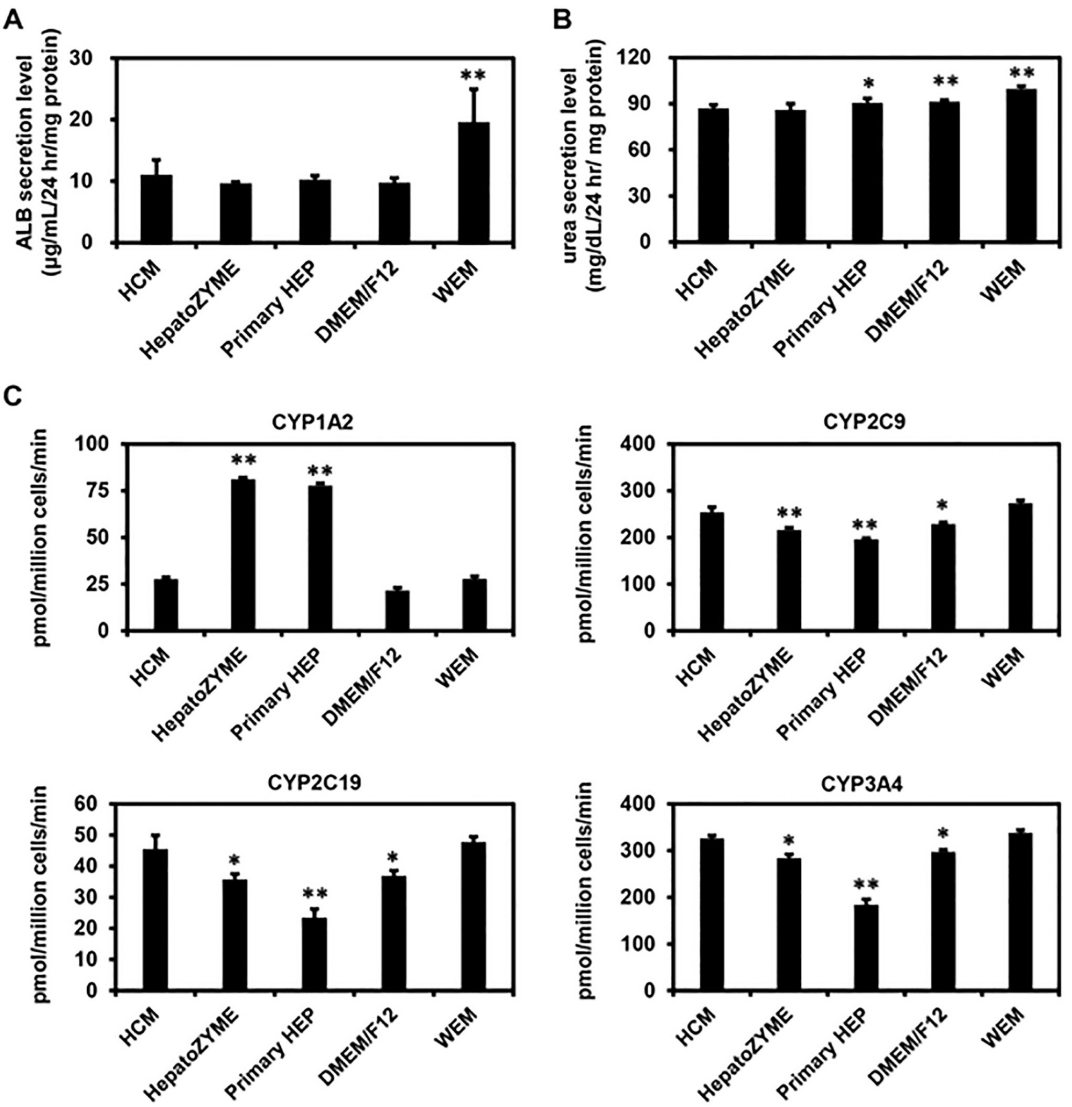
Functional analysis of primary human hepatocytes maintained with various culture media. (**A**, **B**) The ALB (**A**) and urea (**B**) secretion capacities in PHH were examined. (**C**) The CYP1A2, CYP2C9, CYP2C19, and CYP3A4 activities in PHH were examined using LC-MS/MS. Results are shown as the mean ± SD (*n* = 3). Statistical significance was evaluated by one-way ANOVA followed by Dunnet’s test post-hoc tests (compared with “HCM” group). *p < 0.05, **p < 0.01.

Finally, we examined which medium was the most suitable for the maintenance of PHH. PHH were cultured for 90 hr in different media according to the protocol shown in Fig 6A. The phase images of PHH cultured in the different media are shown in Fig 6B. There was little difference in the gene expression levels of hepatocyte markers (*αAT*) among the PHH cultured in the different media (Fig 6C). We also performed a functional analysis of the PHH maintained in the various culture media. The ALB secretion capacity of PHH was the highest in the culture using WEM (Fig 7A). However, there was little difference in the urea secretion capacity among PHH cultured in the different media (Fig 7B). In addition, the CYP2C9, CYP2C19, and CYP3A4 activities in human iPS-HLCs were the highest when HCM or WEM was used (Fig 7C). These results suggest that HCM and WEM were the most suitable media for the maintenance of PHH.

## Discussion

In this study, we compared five media (HCM, HepatoZYME, Primary HEP, DMEM/F12 and WEM) to examine which was the most suitable for culture of human iPS-HLCs and PHH. We measured the ALB secretion levels and CYP activities in human iPS-HLCs and PHH. We found that the hepatocyte culture medium had the strongest influence on the hepatic differentiation efficiency and hepatic functions of human iPS-HLCs and PHH.

The results shown in Figs 1 and 2 suggest that HCM and WEM were the most suitable media for hepatic differentiation of human iPS cells based on the percentages of αAT-positive cells, the amounts of ALB secretion, and the activities of CYPs. However, for the maintenance of human iPS-HLCs, HCM was suggested to be a more suitable medium than WEM (Figs 3 and 4). Tomizawa et al. have developed a culture medium that can purify embryonic stem cell-derived hepatoblast-like cells. They reduced the content of glucose and pyruvate on the medium to adapt hepatic typical way of metabolism [16]. This report suggested that there is a difference in cell metabolism between the hepatic differentiation process and hepatic maintenance process. Therefore, the optimal medium for the hepatic differentiation process might be different from that for the maintenance process. In addition, the activities of CYP1A2 in human iPS-HLCs and PHH cultured with HepatoZYME or Primary HEP were higher than those in human iPS-HLCs and PHH cultured with the other media. Because the compositions of HepatoZYME and Primary HEP have not been published, it might be assumed that these media may contain some inducer of CYP1A2 such as omeprazole. To examine whether HepatoZYME or Primary HEP contain a CYP1A2 inducer, it is necessary to evaluate the CYP1A2 induction potency. In Fig 5, it was shown that the hepatic functions of human iPS-HLCs can be maintained for 3 weeks using HCM. Therefore, it is expected that human iPS-HLCs can be applied to long-term drug toxicity tests. PHHs show a rapid decline in hepatic functions after the plating, thus human iPS-HLCs may be more suitable for long-term drug toxicity tests.

In this study, five media were compared to determine which was the most suitable for culturing PHH and human iPS-HLCs. The results showed that the hepatic functions of human iPS-HLCs and PHH were largely affected by the hepatocyte culture medium. Therefore, it is necessary to explore the components that have a positive effect on the hepatic functions of human iPS-HLCs and PHH. Such components will be useful in promoting maturation of human iPS-HLCs and maintaining the functions of PHH. In this study, we focused on human iPS-HLCs and PHH, and performed a screening for the most suitable medium for the culture of human iPS-HLCs and PHH. Recently, primary hepatocytes isolated from human liver chimeric mice and human hepatoma cell lines have also been used as human hepatocyte models. Therefore, it is expected that more general knowledge can be obtained by performing similar screenings in other human hepatocyte models. By selecting an optimal hepatocyte model and its most suitable culture medium, we will be able to carry out toxicity screening for drug discovery with high accuracy and high sensitivity.

## Supporting information

S1 File (PDF)

## Abbreviations

αAT: alpha-1antitrypsin
AFP: alpha-fetoprotein
ALB: albumin
BMP: bone morphogenetic protein
CAR: constitutive androstane receptor
CYP: cytochrome P450
FGF: fibroblast growth factor
HCM: hepatocyte culture medium
HGF: hepatocyte growth factor
HLCs: hepatocyte-like cells
iPS: induced pluripotent stem
OsM: oncostatin M
PHHs: primary human hepatocytes
WEM: william’s E medium
