# Supplementary material for: Comparison of commercially available media for hepatic differentiation and hepatocyte maintenance
Source: PLoS One. 2020 Feb 27;15(2):e0229654. doi: 10.1371/journal.pone.0229654 (PMC7046223; doi:10.1371/journal.pone.0229654)
Supplement: S1 File — (PDF) [file pone.0229654.s001.pdf]

Title

Comparison of commercially available media for hepatic differentiation and hepatocyte maintenance

Authors

Yukiko Toba, Sayaka Deguchi, Natsumi Mimura, Ayaka Sakamoto, Kazuo Harada, Kazumasa Hirata, Kazuo Takayama, Hiroyuki Mizuguchi

INVENTORY OF SUPPORTING INFORMATION FILE S1

Table A
Table B
Table C

Supplemental tables

Table A Primers used for real-time RT-PCR

Gene Symbol	for real time RT-PCR<br>Primers (forward/reverse; 5' to 3')
aAT	AAGGAGAGAACACTGCTCGTG/CATGCCTAAACGCTTCATCA
AFP	TGGGACCCGAACTTTCCA/GGCCACATCCAGGACTAGTTTC
ALB	GCACAGAATCCTTGGTGAACAG/GCACAGAATCCTTGGTGAACAG
CAR	AGATGCCTTTAGGTCCAATGGG/AGATGCCTTTAGGTCCAATGGG
CYP3A4	AAGTCGCCTCGAAGATACACA/AAGGAGAGAACACTGCTCGTG
GAPDH	GGTGGTCTCCTCTGACTTCAACA/GTGGTCGTTGAGGGCAATG

Table B Antibodies used for Immunocytochemistry

Antigen	Type	Company	Catalog number	Dilution factor
αAT	rabbit	Dako	A0012	1:200
Alexa Fluor 488 anti-rabbit IgG	donkey	Life Technologies	A21206	1:1000

Table C Antibodies used for FACS analysis

Antigen	Type	Company	Catalog number	Dilution factor
αAT	rabbit	Dako	A0012	1:200
Alexa Fluor 488 anti-rabbit IgG	donkey	Thermo Fisher Scientific	A21206	1:1000
